# Multimorbidity and the need to rethink Medical Education

**DOI:** 10.1177/26335565231207811

**Published:** 2023-10-15

**Authors:** Cara Bezzina, Lindsey Pope

**Affiliations:** 1 3526 University of Glasgow, Glasgow, UK

**Keywords:** Medical education, curriculum, assessment, multimorbidity, health professions education

The prevalence of patients experiencing multimorbidity is increasing year on year, accompanied by an expanding knowledge base in this field. Perhaps once considered the domain of geriatricians and general practitioners, all healthcare professionals are now likely to encounter, assess and manage patients living with multimorbidity as part of their daily practice. Despite our recognition and understanding of this changing profile of our patients, the evidence on how best to educate our healthcare workforce to meet the population’s needs lags behind. We propose that all healthcare education and training must give due consideration to the positioning of multimorbidity in their curricula and assessments. Furthermore, expertise from both educationalists and multimorbidity researchers will be required to inform key decisions.

## The challenge: The relationship between curriculum and assessment

While clinician educators acknowledge that we are educating students for more than just passing exams, it would be naïve to consider the role of the curriculum on learning without considering the impact of assessment on what is learnt. Educators can spend hours designing what appear to be high-quality curricula, but these efforts can easily be devalued if this content is not properly assessed. What is taught does not automatically translate into what is learnt, as learners often strategically prioritise what they think is likely to be examined.

Multimorbidity does not fit as readily as single disease presentations into traditional assessment blueprints. For example, this is evident in reviewing the Content Map for the new UK Medical Licensing Assessment (MLA), which will be undertaken by medical students wishing to join the UK register from 2025 onwards.^1^ While the importance of long-term conditions is recognised, the term ‘multimorbidity’ is not included. This omission is surprising given the highlighted need for newly qualified doctors to *‘be able to care for growing numbers of patients with multiple morbidities’* in the General Medical Council (GMC) *Outcomes for Graduates* guidance, which outlines what all graduating UK doctors need to know.^2^

This issue is mirrored in other countries. An analysis of the Medical Council of Canada's Qualifying Examination Objectives,^3^ reveals that those pertaining to the *Medical Expert* focus solely on individual diseases or presentations. In contrast, there is recognition of the importance of caring for patients with ‘multiple medical problems’ in primary care postgraduate curricula, such as The College of Family Physicians of Canada’s Assessment Objectives.^4^ However, as previously stated, patients with multimorbidity are not solely cared for in primary care. While there is a growing body of evidence internationally on clinical reasoning in the context of multimorbidity,^5^ there needs to be greater acknowledgment and integration across medical education.

So, why the apparent disconnect between the curriculum and assessments? The impact and limitations of the chosen assessment modalities needs to be considered**.** A combination of Single Best Answer (SBA) and Objective Structured Clinical Examination (OSCE) style assessments dominate in medical education. In contrast, workplace-based assessments (e.g., case-based discussions and portfolio entries), though contributing to overall progression decisions, are often seen as less valuable as they primarily serve as formative assessments. Instead, high-stakes summative assessments repeatedly present case vignettes with a ‘single best answer’. While these assessments are perceived as robust evaluations of students’ medical knowledge, there may be an unintended consequence that we teach that there is always a ‘right answer’ and that a single disease focus is the best way to approach a patient. Similarly, the drive for standardisation and reproducibility in OSCEs can come at the price of reduced authenticity and assessment of vital complex diagnostic and management skills.

## The solution: The relationship between generalists, education and research

Given the prevalence of multimorbidity, it is inevitable that students will encounter patients with ‘multiple medical problems’ during their placements. However, it is often not highlighted or prioritised in the clinical learning environment. The NICE Guidance on Multimorbidity highlights the need to adopt a clinical approach that accounts for multimorbidity.^6^ Indeed, it has been acknowledged that all graduating doctors will need to acquire and develop the generalist skills to meet the future population's needs.^7^ The value of generalism must be recognised in curricula and by institutions if we are to prepare the next generation of health professionals to better address the challenges posed by multimorbidity. We are beginning to see this embraced by some organisations but not all. A recent positive example is Health Education England’s Enhancing Generalist Skills programme.^8^

How we assess the acquisition and development of these skills also needs broader consideration. While there are complementary assessment modalities suitable for assessing the necessary generalist skills to manage a patient with multimorbidity, how these are represented and valued within curricula needs to be critiqued. These decisions are shaped not only by curriculum and policy documents but also by the values of those in key educational leadership roles. National assessments can be a tool to shape medical school curricula, but it could be argued that ensuring a balance of both generalists and specialists informing key decisions is just as critical given the traditional hierarchical nature of medical schools.

A final piece in the puzzle is the relationship between research and health professions education. In recent years, many universities have decoupled the research and education elements of academia. We would propose that the opposite is required to optimise our students’ learning. As our knowledge of the management of multimorbidity at an individual and population level has grown, so has the field of health professions education. Working synergistically to draw on the latest evidence in both fields we can facilitate the development and evaluation of evidence-informed education. The ability to critically review what is learnt is as crucial as describing current best clinical practice to learners. Only through this joint approach can we build the evidence base needed to pave the way for delivering education that will lead to better outcomes for our patients living with multimorbidity.

## ORCID iD

Cara Bezzina https://orcid.org/0000-0003-4897-0850
